# Genome-Wide Transcriptome Profiling Provides Insight on Cholesterol and Lithocholate Degradation Mechanisms in *Nocardioides simplex* VKM Ac-2033D

**DOI:** 10.3390/genes11101229

**Published:** 2020-10-20

**Authors:** Victoria Y. Shtratnikova, Mikhail I. Schelkunov, Victoria V. Fokina, Eugeny Y. Bragin, Tatyana G. Lobastova, Andrey A. Shutov, Alexey V. Kazantsev, Marina V. Donova

**Affiliations:** 1 Belozersky Research Institute of Physico-Chemical Biology, Lomonosov Moscow State University, Moscow 119991, Russia; 2 Skolkovo Institute of Science and Technology, Moscow 121205, Russia; m.shchelkunov@skoltech.ru; 3 Institute for Information Transmission Problems, Russian Academy of Sciences, Moscow 127051, Russia; 4 G.K. Skryabin Institute of Biochemistry and Physiology of Microorganisms, Federal Research Center “Pushchino Center for Biological Research of the Russian Academy of Sciences”, Pushchino142290, Russia; 2vvfokina@gmail.com (V.V.F.); bragory@yandex.ru (E.Y.B.); lobastova_t@rambler.ru (T.G.L.); w___w@rambler.ru (A.A.S.); donova@ibpm.pushchino.ru (M.V.D.); 5 Chemistry Department, Lomonosov Moscow State University, Moscow 119991, Russia; mak@org.chem.msu.ru

**Keywords:** bacterial transcriptomics, NGS, steroid bioconversion, transcriptional factors, actinobacteria

## Abstract

Steroid microbial degradation plays a significant ecological role for biomass decomposition and removal/detoxification of steroid pollutants. In this study, the initial steps of cholesterol degradation and lithocholate bioconversion by a strain with enhanced 3-ketosteroid dehydrogenase (3-KSD) activity, *Nocardioides simplex* VKM Ac-2033D, were studied. Biochemical, transcriptomic, and bioinformatic approaches were used. Among the intermediates of sterol sidechain oxidation cholest-5-en-26-oic acid and 3-oxo-cholesta-1,4-dien-26-oic acid were identified as those that have not been earlier reported for *N. simplex* and related species. The transcriptomic approach revealed candidate genes of cholesterol and lithocholic acid (LCA) catabolism by the strain. A separate set of genes combined in cluster and additional 3-ketosteroid Δ^1^-dehydrogenase and 3-ketosteroid 9α-hydroxylases that might be involved in LCA catabolism were predicted. Bioinformatic calculations based on transcriptomic data showed the existence of a previously unknown transcription factor, which regulates cholate catabolism gene orthologs. The results contribute to the knowledge on diversity of steroid catabolism regulation in actinobacteria and might be used at the engineering of microbial catalysts for ecological and industrial biotechnology.

## 1. Introduction

**Figure 1:**
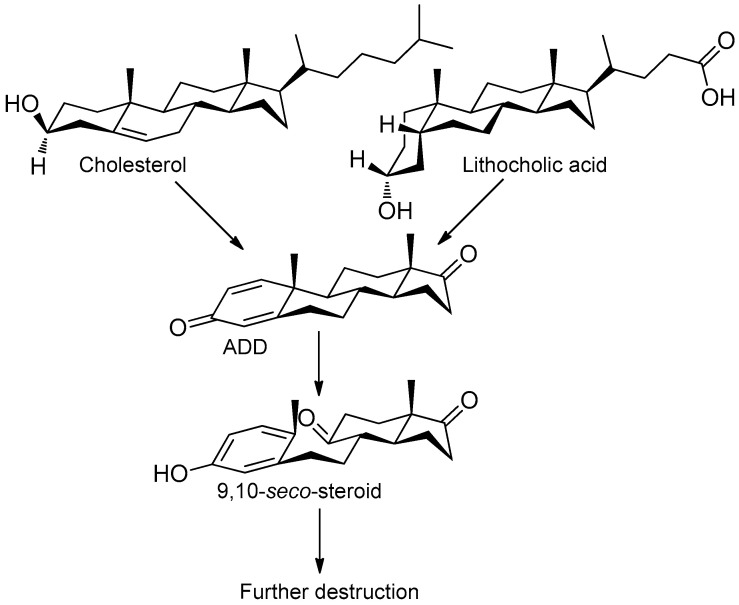
Principal scheme of steroid degradation via androsta-1,4-diene-3,17-dione (ADD) and 9,10-*seco*-pathway. **I**–Cholesterol, **II**–Lithocholic acid (LCA), **III**–ADD, **IV**–9,10-*seco*-steroid.

Sterols, such as cholesterol (**I**), are steroid 3β-alcohols with alkyl side chain at C17 (Figure 1). Due to their unique lipophilic properties, these lipids play vital functions in all living organisms being essential components of the cell membrane that influence membrane fluidity, cell differentiation, and proliferation. In vertebrates, bile acids along with other essential steroids (hormones, fat-soluble vitamins, etc.) are produced from cholesterol via structural modification of the steroid core and shortening of the side chain.

Structurally, cholic acids (**II**) differ from cholesterol **(I)** by *cis*-A/B-ring juncture, α-orientation of hydroxyl at C3, saturated steroid core and a C5 acyl side chain attached to C17 (Figure 1). Unlike other cholic acids, lithocholic acid (LCA, **II**), does not contain any other hydroxyl groups at the steroid core.

Being released into the environment via the decay of biomass or excretion by eukaryotic organisms, cholesterol, LCA, and other steroids may serve as carbon and energy sources for diverse bacteria. Steroid microbial degradation plays a significant ecological role in being a key process for biomass decomposition as well as removal/detoxification of steroid pollutants. Recent metagenome studies highlighted the global distribution of bacteria capable of sterol and cholate degradation with the prevalence of Actinobacteria and Proteobacteria from different habitats (soil, aquatic environments, eukaryotic hosts, and others) [1].

The so-called “9(10)-*seco*-steroid” pathway is the only known route of sterol and cholate degradation by many actinobacteria [2,3,4,5]. Most strains degrade sterols and cholates via 3-keto-1,4-diene structures that are formed due to consecutive oxidations of 3-hydroxy group and introduction of the corresponding double bond(s) into a steroid core (Figure 1).

Approaches based on genetic and metabolic engineering of microbial steroid degraders allow effective production of the value-added steroids by exploiting cascade reactions of the degradative pathway [6,7]. The metabolic engineering of new biocatalysts requires clear insight into the genome organization, gene functions, and regulation.

Over the past decade, much has become known about cholesterol degradation by different actinobacteria such as *Mycobacterium tuberculosis* [8], *Rhodococcus* strains [9,10], *Mycolicibacterium smegmatis* (basonym *Mycobacterium smegmatis*) [11], *Gordonia cholesterolivorans* [12], and proteobacterium *Comamonas testosteroni* [13]. The roles of certain enzymes, mechanisms of their functioning, and interactions with substrates were investigated. Aerobic cholate degradation has been studied mainly for a few *Rhodococcus* strains such as *Rhodococcus jostii* RHA1 [14] and *Pseudomonas* strains such as *Pseudomonas stutzeri* Chol1 [15,16] and *Pseudomonas putida* DOC21 [17].

A soil-dwelling strain of *Nocardioides simplex* VKM Ac-2033D is well-known mainly due to its superior 3-ketosteroid dehydrogenase (3-KSD) activity toward various 3-ketosteroids and effective production of their pharmaceutical 1(2)-dehydro-analogs [18,19,20,21]. The strain is also capable of hydrolyzing steroid esters, reducing C17 and C20 carbonyl groups of androstanes and pregnanes, respectively [18,19,20], transforming deoxycholic acid to 3-keto-deoxycholic acid, and growing on cholic acid [22]. 

In our previous studies, the complete genome of the strain had been sequenced and assembled [23]. Based on the data of bioinformatic screening for steroid catabolism operons, assumptions were made concerning the functionality of a number of genes [24]. In the present work, transcriptome profiling of the strain grown in the presence of either cholesterol or LCA was estimated to support the assumptions. 

In this study, a number of genes were identified that enhance expression in response to cholesterol and LCA, and their functions were suggested. The specific features of cholesterol and LCA degradation by the strain and the corresponding genes involved were investigated in more detail. A number of steroids were detected that had not been reported so far among the intermediates of cholesterol catabolism. Homologs of the genes from the cluster that had been earlier revealed in *Rhodococcus jostii* RHA1 or other actinobacteria as related to cholate degradation [14] were found in *N. simplex*. We confirmed enhanced expression of the genes of the predicted cluster and suggested their functional role in steroid catabolism.

## 2. Materials and Methods 

### 2.1. Reagents

Cholesterol was obtained from Serva (Heidelberg, Germany), LCA–from Acros Organics (Merelbeke, Belgium). Randomly methylated β-cyclodextrin (MCD) was purchased from Wacker-Chemie (Burghausen, Germany), soya peptone–from HiMedia (Mumbai, India), and yeast extract–from Difco (Franklin Lakes, NJ, USA). Other materials and solvents were of an analytical grade and purchased from domestic commercial suppliers.

### 2.2. Microorganism, Cultivation, and Gene Expression Induction

A strain of *Nocardioides simplex* VKM Ac-2033D was obtained from the All-Russian Collection of Microorganisms (VKM) and pre-cultured as described earlier [20].

For an RNA-seq based transcriptomic analyses, the strain was cultured as described earlier [24] in the mineral medium supplemented with (g/L): glycerol–3, yeast extract–3, MCD–1.4 (pH 7.0) (control), and the same medium supplemented with cholesterol (0.2 g/L) or LCA (0.2 g/L) (induction condition) for 16 h.

### 2.3. Growth Estimations

The experiments were carried out in three replicates. Growth on cholesterol vs. LCA as the sole source of carbon was estimated using the mineral medium [20] supplemented with MCD–9.65 g/L. Each steroid was added to the medium up to the final concentration of 1 g/L before sterilization. Cultivation was carried out aerobically on a rotary shaker (220 rpm) at 30 °C for 120 h. For biomass estimation, the samples of the broth were centrifuged, the residue was washed twice with MCD solution (17.8 g/L) to remove steroids, and then the cells were re-suspended in 0.02 K-phosphate buffer (pH 7.0). Lastly, the OD600 of the washed cell suspension was measured. The experiments were performed in triplicates.

### 2.4. Steroid Bioconversion 

Steroid bioconversion was performed in the GSMY (glucose 7 g/L, soluble starch 10 g/L, malt extract 5 g/L, yeast extract 4.5 g/L, and calcium carbonate 0.05 g/L) medium [25]. After 24 h since inoculation, each steroid (cholesterol or LCA) was added as a solution in MCD to a final concentration of 0.5 g/L. Molar ratios (steroid:MCD) were 1:5 or 1:3, for cholesterol or LCA, respectively. Bioconversion was carried out aerobically on a rotary shaker (200 rpm) at 30 °C for 144 h. The experiments were performed in triplicates.

### 2.5. Steroid Metabolite Isolation 

After 6 and 24 h of cholesterol conversion, the biomass was separated from the broth (200–500 mL) by centrifuge (8000× *g*, for 30 min). Steroids were extracted with ethyl acetate (EtOAc), and the combined organic extract was concentrated on a rotary evaporator. Crude residues (25–30 mg) were applied to preparative TLC (thin layer chromatography) plates (ALUGRAM SIL G-200 UV_254_, Macherey-Nagel, Düren, Germany). Individual compounds were eluted with EtOAc and evaporated to dryness. Chromatographic purity of the compounds was controlled by TLC and HPLC.

### 2.6. Thin Layer Chromatography (TLC) 

The TLC plates (ALUGRAM SIL G/UV_254_, Macherey-Nagel, Düren, Germany) were developed in benzene:acetone (4:1, v/v) (System A) and CHCl_3_/acetone/CH_3_COOH 50:50:0.5) (System B), respectively. Cholesterol and its derivatives with 3β-ol-5-ene configuration were visualized under the treatment of the TLC plates with 4% (v/v) phosphomolybdic acid hydrate solution in ethyl alcohol, which was followed by heating to 60–65 °C. Steroids with 3-oxo-4-ene moiety were visualized under UV light (254 nm) using hemiscope CN-15MC UV Darkroom (Vilber Lourmat, Collégien, France). LCA metabolites were assayed as described earlier [22,26].

### 2.7. High-Performance Liquid Chromatography (HPLC) 

Samples for the HPLC analyses were prepared as follows. Aliquots of culture liquid were 20–25 times diluted by acetonitrile-isopropanol solution (50:45, v/v). Suspension was centrifuged at 12,100× *g* for 10 min. Aliquots of the supernatant were used for the analyses. The analyses were performed on the Agilent 1200 instrument (Agilent Technologies, Waldbronn, Germany), column Symmetry C18 (5 µm, 4.6 × 250 mm) with precolumn Symmetry C18 (5 µm, 3.9 × 20 mm) (Waters, Milford, MA, USA). The calibrations were done by an external standard method based on the comparison of peak areas. Processing of results was carried out using ChemStation Rev. B. 04.03 (Agilent Technologies, Santa Clara, CA, USA).

The following conditions were used: flow rate of 1 mL/min, column temperature 50 °C, detection of 3β-ol-5-en/3-keto-4-en configurations at 200/240 nm, respectively, mobile phase for cholesterol and its derivatives: acetonitrile:isopropanol:water (50:45:5 v/v/v) or acetonitrile:water:acetic acid (60:40:0.01 v/v/v).

### 2.8. Mass-Spectrometry (MS), ^1^H-NMR and ^13^C-NMR Spectroscopy

^1^H- and ^13^C-NMR spectra were recorded at 400 and 100.6 MHz, respectively, with a Bruker Avance400 spectrometer. Chemical shifts were measured relative to the solvent signal. Only characteristic signals in ^1^H-NMR of steroids are given. HRMS (high resolution mass spectrometry) experiments were performed with Orbitrap Elite mass-spectrometer (Thermo Fisher Scientific GmbH, Bremen, Germany) with an ESI (electrospray ionization) source.

Mass spectra of the other intermediates were recorded on a Finnigan LCQ Advantage MAX mass spectrometer with a quadrupole ion trap (Thermo Electron, San Jose, CA, USA) in positive ions [M + H]^+^, at the evaporator temperature of 350 °C and capillary temperature of 170 °C. MS/MS (tandem mass spectrometry) spectra were obtained using normalized collision energy (Normalized Collision Energy TM) in the range of 20% to 40%. Data collection and processing was carried out using Xcalibur v.1.3 software.

### 2.9. Isolation of mRNA and High-Throughput Sequencing

The cells taken in the exponential growth phase (16 h) were harvested by centrifuge at 8000× *g* for 10 min. mRNA was isolated as described earlier [27]. The resulting mixture was treated with a Turbo DNA-free kit (Thermo Fisher Scientific, Whaltham, MA, USA). Library preparation of mRNA for high-throughput sequencing was made with R3000 Zymo-Seq RiboFree Total RNA Library Kit (Zymo Research, Irvine, CA, USA). This kit provided the majority of CDS (coding sequences)-aligned reads. 

Sequencing was conducted on HiSeq 4000 according to the protocols of the manufacturer (Illumina, San Diego, CA, USA). Sequencing reads are deposited in the NCBI Sequence Read Archive as a part of the project “Study of bacteria which catabolize steroids” (https://www.ncbi.nlm.nih.gov/bioproject/PRJNA350502).

Real-time PCR validation was performed with using the AriaMx Realtime PCR system (Agilent, Richardson, TX, USA) with an M-439 kit (Eva Green I) (Syntol, Moscow, Russian Federation). The nucleotide sequences of primers used in this study for target and reference genes are listed in Table S1. The amplification was performed as follows: 95 °C 5 min (1 cycle), 95 °C 10 s, 54 °C 15 s, 72 °C 30 s (40 cycles). Relative gene expression levels were calculated using the double delta Cq method [28].

### 2.10. Computational Analyses

Adapter trimming was performed by Trimmomatic 0.39 [29]. Reads that became shorter than 30 bp after the trimming were discarded. The differential expression analysis was done by Rockhopper 2.0.3 [30]. No more than 2% of bases per read were allowed to differ from the reference sequence during the mapping stage (“-m 0.02”). Operons were also predicted by Rockhopper. 

Orthologous and paralogous relations between genes of *N. simplex* VKM Ac-2033D, actinobacteria, and anaerobic intestinal bacteria were analyzed using the OrthoFinder [31]. The strains with known complete and annotated genome were chosen (Table S2). Proteins of the selected strains were clustered into orthologous groups (orthogroups). 

Genes whose products could play a role in the biochemical reactions were predicted based on the annotation, homology with the known enzymes (using orthogroups), gene expression changes, location, and predicted regulons to which the orthologs belonged.

### 2.11. Search of Transcription Factor Binding Motifs

To find motifs for possible regulators of the LCA catabolism, the following procedure was used. All the genes increased their expression at least threefold when LCA-induced as compared to the control with a maximum *q*-value of the change set at 0.05. Regions 500 bp upstream and 50 bp downstream (totaling 550 bp, “550 bp regions”) with respect to the start codons of all such genes were extracted from the genome sequence. Short sequences overrepresented in these 550 bp regions were searched using the software MEME from MEME Suite 5.1.1 [32] with the following settings: (a) motifs should be between 8 bp and 50 bp long, (b) any number of sites was allowed in each of the 550 bp regions, (c) the *e*-value of a motif should not exceed 0.1, and (d) there was no mandatory requirement for a motif to be palindromic. To test whether the motifs found in the previous step could belong to the transcription factors that regulate LCA catabolism, we scanned 550 bp regions of all *N. simplex* VKM Ac-2033D genes for these motifs by FIMO from MEME Suite 5.1.1 with maximum *q*-values of sites set at 0.05. The nucleotide frequencies in the analyzed regions were calculated by the tool Fasta-get-markov from MEME suite and utilized by FIMO using the option “--bfile”. 

A motif was considered as potentially belonging to a transcription factor regulating the LCA catabolism if the FIMO results for that motif satisfied two criteria simultaneously. Criterion A: no more than 1000 sites of that motif were found. If such a large number of sites were indicated by FIMO, the motif likely belonged to the promoter sequence or some widespread low-complexity sequences like microsatellites. Criterion B: more than 10% of sites of that motif were located in 550 bp regions of the first genes of operons that contained at least one gene that significantly (*q*-value ≤ 0.05) increased its expression in response to LCA. Though our knowledge of operon borders is imprecise, this criterion increases the probability that the motif belongs to a transcription regulator of the LCA catabolism.

An additional technique based on phylogenetic footprinting was used to find motifs of transcription regulators for genes *KR76_17985–KR76_18085* (cluster E, see Results 3.3) that could be missed by the previous method. Sequences from homologous bacteria were used to increase the number of target regions provided to MEME, which, in turn, may increase the sensitivity of motif detection. Motifs of KstR and KstR2 are very similar between *N. simplex* and *Mycobacteria* [24], which suggests the feasibility of phylogenetic footprinting in our case. The methodology was as follows.

(1) There were eight operons in cluster E with at least one gene that significantly changed its expression. The first genes of these operons were taken and their proteins were aligned by BLASTP from BLAST 2.9.0+ [33] to proteins from all actinobacterial genomes in NCBI RefSeq. The RefSeq database was current as of 16 May 2020 and contained 1307 actinobacterial genomes. The BLASTP alignment was done with the *e*-value threshold of 10^−3^ and all other parameters set as default. 

(2) For each of the eight proteins, twenty homologs were taken with the highest *e*-values. Along with the eight proteins of *N. simplex*, this produced a set of 168 proteins. 550 bp regions were taken for the genes of all these proteins. 

(3) Motifs in those 168 regions were predicted by MEME with the same options as described above. 

(4) Sites of those motifs were searched by FIMO in 550-bp regions of all *N. simplex* genes with the same options as described above. 

(5) Since the motifs created in step 3 were based not only on sequences from *N. simplex*, but also from other actinobacteria, we recalculated the motifs based only on the sites found by FIMO. Then, with these updated motifs, we scanned the 550 bp regions of *N. simplex* by FIMO again. This operation lessens the contribution of actinobacteria other than *N. simplex* to the motifs. 

(6) If the sites found for a motif in steps 4 or 5 failed to satisfy criterion A or criterion B, the analysis of the motif was aborted as it was unlikely to belong to a transcription factor regulating the catabolism of the LCA.

Logos of motifs were built using WebLogo2.8.2 [34].

## 3. Results

### 3.1. Growth on Cholesterol and LCA

**Figure 2:**
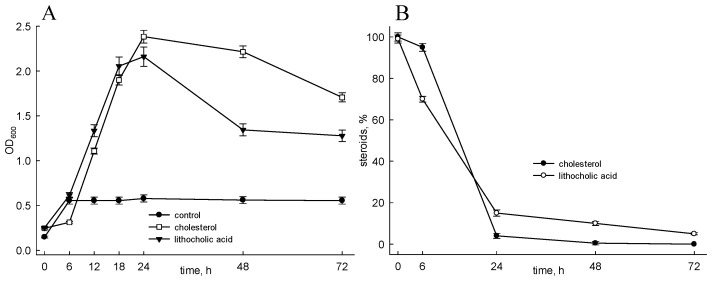
Growth of *Nocardioides simplex* on cholesterol or LCA and consumption of substances. (**A**) Growth of *N. simplex* on cholesterol or LCA. (**B**) Consumption of cholesterol, or LCA during *N. simplex* growth. Mineral medium supplemented with MCD was used in the control. Cholesterol, or LCA were added to the medium as per (2.3). Steroids were assayed by TLC as per (2.6). The data are the average of triplicates.

As shown in Figure 2A, the strain is able to grow on cholesterol as well as LCA as sole carbon and energy sources, and both substrates were almost fully utilized for 72 h under the conditions used (Figure 2B). No growth or very poor growth was observed in the control (mineral medium supplemented with MCD but without steroids) (Figure 2A).

### 3.2. Cholesterol and LCA Bioconversion 

Under the cholesterol bioconversion conditions, a number of 3-keto-4-ene-steroids were detected in the broth, such as cholest-4-en-3-one (**V**), cholesta-1,4-dien-3-one (**VI**), 26-hydroxy-cholest-4-en-3-one (**VII**), 3-oxo-cholest-4-en-26-oic acid (**VIII**), and 3-oxo-cholesta-1,4-dien-26-oic acid (**IX**) (Table 1). Moreover, the intermediate with the preserved 3β-hydroxy-5-ene-configuration was revealed in minor amounts, namely, 3β-hydroxy-cholest-5-en-26-oic acid (**X**) (Table 1). The characteristics of the compounds are given in Table S3.

No metabolites or intermediates were revealed during LCA bioconversion by the *N. Simplex* Data on cholesterol or lithocholic acid bioconversion (substrates and intermediates) are shown in Figure S1 (TLC).

### 3.3. Transcriptome Sequencing Differently Expressed Genes

The differential expression of genes was estimated for strain growth on cholesterol or LCA as compared to the medium without any steroids. The statistical data of the sequencing results are shown in Table S4. Reads are deposited as the NCBI Sequence Read Archive (https://www.ncbi.nlm.nih.gov/bioproject/PRJNA350502).

**Figure 3:**
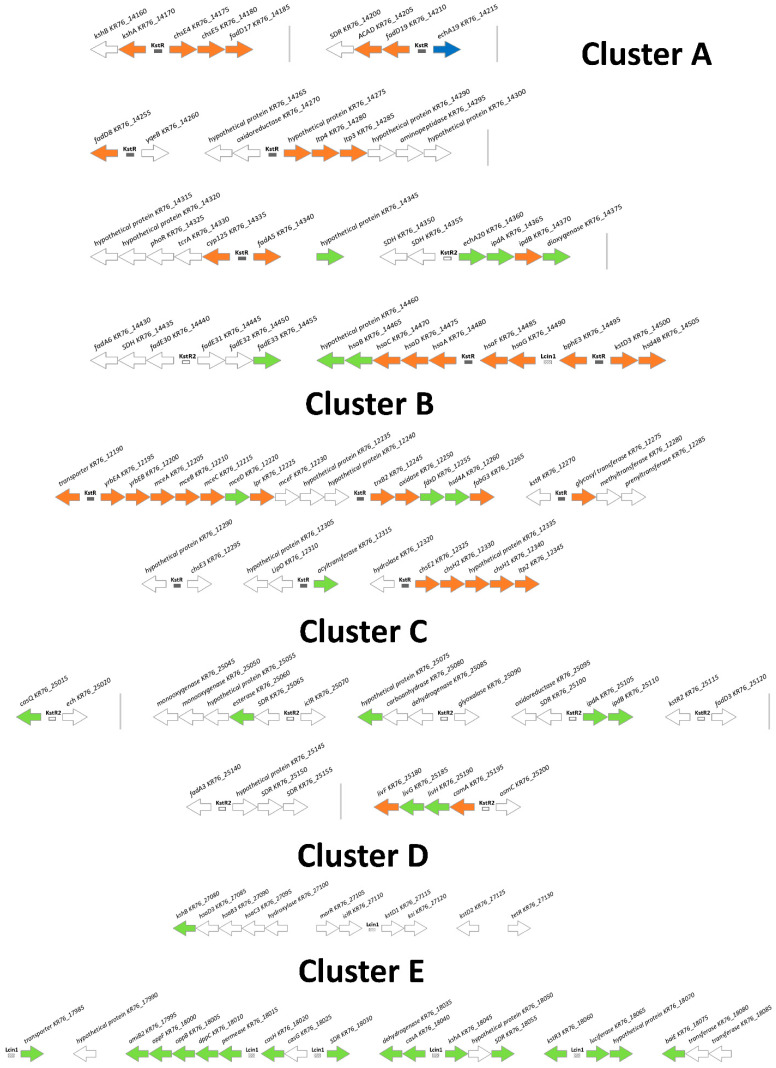
Clusters of *N. simplex* VKM Ac-2033D genes coding for steroid metabolism related proteins. Genes are ordered from top left to bottom right. Vertical lines designate regions where several genes are omitted. Neighboring operons are separated by a larger space. Filled rectangles denote predicted KstR binding sites, empty rectangles denote predicted binding sites of KstR2, rectangles with diagonal shading—predicted Lcin1 binding sites. White arrows indicated genes with no observed expression increase, blue arrows—genes with expression increased on medium with cholesterol, green arrows—genes with expression increased on medium with LCA, orange arrows—genes with expression increased on both inductors.

**Figure 4:**
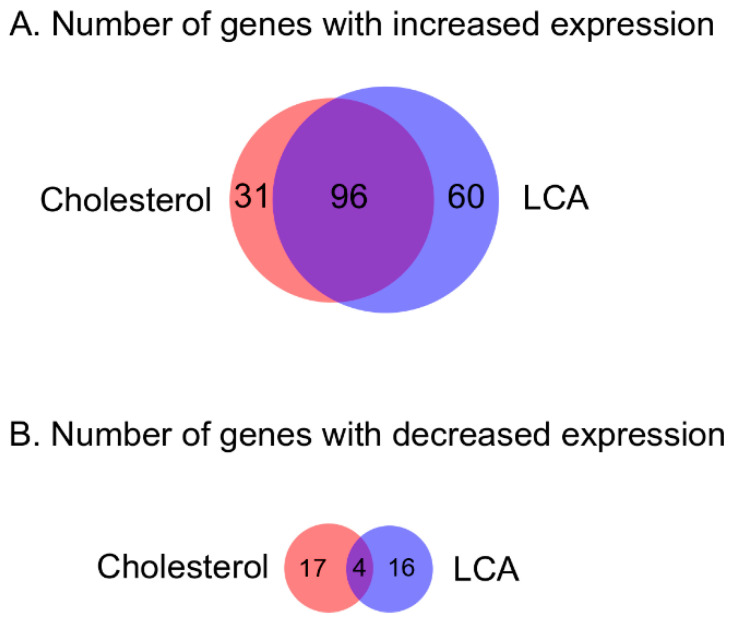
Venn diagram for the *N. simplex* VKM Ac-2033D with the number of genes that changed expression in response to cholesterol or LCA. (**A**) Genes with increased expression. (**B**) Genes with decreased expression.

**Figure 5:**
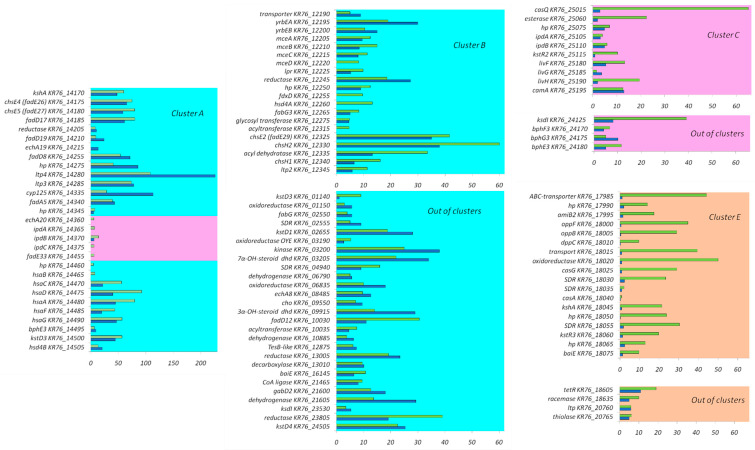
Differentially expressed genes in the *N. simplex* VKM Ac-2033D genome. Blue bars–expression induction by cholesterol (fold change), green bars–expression induction by LCA (fold change), light blue color–predicted KstR-regulon, pink–predicted KstR2-regulon, light orange–predicted Lcin1-regulon. Only genes that changed expression more than threefold with *q*-value less than 0.05 are shown. Abbreviations: hp–hypotetical protein, dhd–dehydrogenase, SDR–short-chain dehydrogenase-reductase. X-axis–expression changes (for cluster A the scale is expanded to 200).

The genes induced by the steroids formed several clusters as reported earlier [24] (Figure 3), and separate induced genes were also revealed. In total, 127 genes enhanced their expression in response to cholesterol, and 156 genes in response to LCA (Table 2, Figure 4). Levels of induction of DEGs (differentially expressed genes) are shown in Figure 5. In particular, LCA activated gene transcription in cluster E (Table 2). Genes were combined into operons according to the previous bioinformatics studies [24] and the transcriptomic data. Based on the expression, the previously predicted boundaries of cluster E were expanded to include highly expressed operons nearby. The full list of the operons with DEGs is shown in Table S5. Real-time validation results for three genes are shown in Table S1.

To study orthologous and paralogous relations between the genes of *N. simplex* VKM Ac-2033D, actinobacteria and anaerobic intestinal bacteria gene orthogroups were built. The list of the orthogroups (Table S2) was used for gene function prediction.

### 3.4. Candidate Motifs for Transcriptional Regulators of LCA Catabolism in N. Simplex

The search for motifs of transcription factors that could regulate LCA catabolism in *N. simplex* with MEME resulted in three motifs that passed the filtering criteria. Two of these motifs coincided with the motifs described earlier for KstR and KstR2 [24], and the third one (named Lcin2) will be described below.

Simple searching of transcription factor motifs with MEME was not able to find previously unknown motifs of the transcription factors that regulated cluster E. To find such motifs, an alternative approach, phylogenetic footprinting, was used. The phylogenetic footprinting analysis suggested that there were two motifs in cluster E. One of them belonged to KstR and had been reported earlier [24]. The presence of the second motif (Lcin1, Lcin is an abbreviation for LitoCholate INcrease) was indicated by MEME with an *e*-value of 1.2 × 10^−191^. Its logo is represented in Figure S2a. The consensus of Lcin1 is a perfect palindrome TCCCGGT[C/G]ACCGGGA. Genes with sites of Lcin1 (primarily, genes from cluster E) are shown in Figure 3.

The third motif found by MEME was called Lcin2. Its logo is depicted in Figure S2b. The *e*-value for the existence of this motif is 5 × 10^−8^. Lcin2 is non-palindromic. It has six corresponding sites in the genome including five that are located before operons that have DEGs in LCA plus conditions (Table S5). Among others, sites of this motif are found in the 550bp regions for genes *KR76_14335* (*cyp125*), *KR76_14340* (*fadA5*), and *KR76_18605* (TetR transcriptional regulator).

The full list of the genes with revealed sites is shown in Table S5.

## 4. Discussion

### 4.1. Clusters and Regulons

In our previous study [24], the clusters of the genes homologous to the known steroid catabolism genes have been predicted in the genome of *N. simplex* VKM Ac-2033D based on the correspondence of the operons with the operons previously studied in other works. Cluster A includes the genes of KstR-regulons and KstR2-regulons involved in steroid side chain oxidation and different steps of steroid core degradation including ring B cleavage, ring A oxidation, and rings C and D degradation. Cluster B contains KstR-regulon genes related to a side chain β-oxidation pathway. According to the bioinformatic predictions, cluster C belongs to the KstR2-regulon and is most likely involved in the lower degradation pathways, i.e., final steps of ring B destruction. The genes of cluster D, by the homology, are related to ring B cleavage and ring A destruction. However, neither KstR-binding nor KstR2-binding sites were found in the promoters of cluster D genes. Presumably, these operons might be activated by some steroids that structurally differ from cholesterol or LCA.

Aerobic cholate degradation has much in common with the 9(10)-*seco* pathway of steroid catabolism [2]. Similar to that in other steroid transforming actinobacteria, most of the *N. simplex* genes related to both cholesterol and LCA degradation are located in the conserved clusters A, B, and C, and their expression is predicted under repression control of the transcriptional regulators KstR and KstR2. Transcription of the genes was activated in LCA and cholesterol grown cells (Figure 3 and Figure 5). As shown for *Rhodococcus jostii* RHA1, the genes involved in the cholate degradation group is in a separate cluster [14]. A number of homologous genes of this cluster have also been identified in the genome of *N. simplex* VKM Ac-2033D. The closely located operons of these genes can be combined into a separate cluster (cluster E) [24]. Expectedly, most of the genes in this cluster significantly increased their expression in response to LCA (Figure 3), but not cholesterol. In addition, we found a sharp increase in the expression of an additional ortholog of 3-ketosteroid dehydrogenase *KR76_01140* located outside the clusters.

### 4.2. Initial Steps of Cholesterol Oxidation

**Figure 6:**
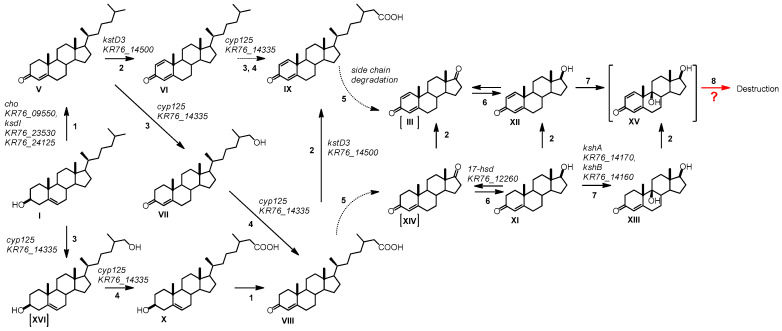
Cholesterol side chain oxidation by *N. simplex* VKM Ac-2033D. Substances: **I**-Cholesterol, **V**-Cholest-4-en-3-one, **VI**-Cholesta-1,4-dien-3-one, **VII**-26-Hydroxy-cholest-4-en-3-one, **VIII**-3-Oxo-cholest-4-en-26-oic acid, **IX**-3-Oxo-cholesta-1,4-dien-26-oic acid, **X**-3β-Hydroxy-cholest-5-en-26-oic acid, **XI**-Androst-4-en-17β-ol-3-one, **XII**-Androsta-1,4-dien-17β-ol-3-one, **XIII**-Androst-4-ene-9α,17β-diol-3-one, **XIV**-Androst-4-ene-3,17-dione, **III**-Androsta-1,4-diene-3,17-dione, **XV**-Androsta-1,4-dien-9α,17β-diol-3-one, **XVI**-26-Hydroxy-cholesterol. Compounds in square brackets were not found among the bioconversion products under the indicated conditions. Biochemical reactions: 1–3β-Hydroxyl group dehydrogenation and ∆^5→^∆^4^-isomerization,2–3-Oxo-4-ene-steroids 1(2)-dehydrogenation,3–C26-Methyl group hydroxylation,4-C26-Alcohol hydroxylation,5–Oxidative side-chain degradation,6–17β-Reduction/oxidation; 7-9α-Hydroxylation,8–Ring B cleavage with *seco*-derivative formation. Locus tags in *N. simplex* are predicted.

Based on the detected intermediates and dynamics of the intermediate accumulation, the following scheme of cholesterol bioconversion with *N. simplex* VKM Ac-2033D cells was created (Figure 6).

As shown in Figure 6, the initial reaction is transformation of cholesterol (**I**) to cholest-4-en-3-one (**V**). It is well established that the reaction is catalyzed either by cholesterol oxidases or 3-hydroxysteroid dehydrogenases (3-HSD) (see Reference [35] for review). Cholesterol oxidase encoded by *KR76_09550* seems to be involved in this reaction in *N. simplex* (Table 3). Distant homologs of the genes coding for ChOs with a covalently bound FAD cofactor such as ChO (AAF64503) from *Pimelobacter simplex* [36], namely, ChO encoded by *KR76_07670* were also identified in the genome of *N. simplex* as well as ChO with a noncovalent cofactor binding such as ChOs from *Mycobacterium leprae*, *Salinispora arenicola*, and *Rhodococcus equi* [37] namely, *KR76_01200*, *KR76_06800*, and *KR76_13200*. None of the previously mentioned genes except for *KR76_09550* was induced by cholesterol, or LCA in *N. simplex*. No orthologs of the extracellular *choG* from *Rhodococcus erythropolis* [36] or *Rhodococcus ruber* Chol-4 [38] was found in *N. simplex*. Moreover, no genes with high identity to the 3-HSDs have been revealed in the genome of *N. simplex*. 

Among the genes that activate transcription in response to cholesterol and LCA, the gene *KR76_24505* (Table 4) was revealed as orthologous to *kst4D* of *R. jostii* RHA1 [47]. Similar to the known Kst4Ds from other steroid-transforming actinobacteria, it may be active toward 5α-3-ketosteroids such as LCA bioconversion intermediates and catalyze *trans*-axial elimination of the 4β-hydrogen as a hydrogen ion to a nucleophilic residue of the active site through a protein-based reaction and the 5α-hydrogen as a hydride ion to the *N*(4)-position of the enzyme-flavin. Kst4D has a wide specificity for 3-keto-5α-steroids, and 3-keto-1-ene-steroids were shown to be more preferable substrates than the corresponding 1,2-saturated steroids [35,48]. However, the role of Kst4D in cholesterol degradation by *N. simplex* requires further investigation. In *Comamonas testosteroni*, ATCC 17,410 and TA441, the *kstD4* gene is located downstream of *kstD* (*tesI* and *tesH* in TA441), and both genes, forming an operon, are co-expressed [35,49,50,51]. In contrast, the gene *KR76_24505* in *N. simplex* is itself a transcriptional unit.

Cholest-4-en-3-one (**V**) is further transformed to cholesta-1,4-dien-3-one (**VI**) followed by its 26-hydroxylation to form 3-oxo-cholesta-1,4-dien-26-oic acid (**IX**) (Figure 6). Detection of **VI** and **IX** among the intermediates confirms that 1-dehydrogenation occurs at the early steps of cholesterol oxidation in *N. simplex*. To the best of our knowledge, these steroids have not been reported so far, as intermediates of the cholesterol degradation pathway in *N. simplex* and related species. Moreover, 3-ketosteroid dehydrogenases (3-KstD) have been known to prefer 3-oxosteroids without bulky side chains at C-17 as substrates [52]. Thus, 1-dehydrogenation of cholestane steroids could be considered as an exceptional feature of *N. simplex* VKM Ac-2033D. As reported earlier, at least five 3-ketosteroid dehydrogenase isoforms were revealed in the genome of this strain [24]. The possible role of KstD3 coded by *KR76_14500* in cholesterol intermediate 1-dehydrogenation (**V**→**VI**) was proposed (Table 3). In parallel to the oxidation of the 3β-hydroxyl group, hydroxylation at C26 occurred, thus, resulting in the 3β-hydroxy-cholest-5-en-26-oic acid (**X**) formation via 26-hydroxy-cholesterol (**XVI**) (Figure 6). Detection of the intermediate with preserved 3β-ol-5-ene-configuration of the steroid core showed that the initial reactions of cholesterol degradation, i.e., modification of 3β-ol-5-ene-moiety and hydroxylation at C26, occurred independently in *N. simplex*. Compounds **X** and **XVI** have not been identified earlier among the intermediates during cholesterol oxidation by *N. simplex* and related actinobacteria. The reaction is most likely catalyzed by Cyp125 known as C-26 hydroxylase [44]. 

The same enzyme might account for the formation of Compound **VII** from **V** and **IX** from **VI** (Figure 6), thus, demonstrating Cyp125 activity towardboth 3-hydroxy steroids, and 3-oxosteroids including those unsaturated at C1(2). As reported earlier, 3-oxo-cholest-4-en-26-oic and their derivatives may play role in the regulation of the transcription factor KstR [53]. Binding this compound together by its 3-oxo-4-ene-moiety and 26-carboxylic group induces conformational changes in KstR, which was followed by KstR release from the operator. The regulatory model also explains the high redundancy of the enzymes involved in these initial steroid degradation steps, including ChOs, 3β-HSDs, KstIs, Cyp125, Cyp142, etc. In *N. simplex*, the gene *KR76_14335* (Table 3, cluster A, predicted KstR-regulon) orthologous to *cyp125* was induced by both cholesterol and LCA.

As mentioned above, neither AD (**XIV**) nor ADD (**III**) have been revealed among the C19-steroids that, though their intracellular formation, cannot be entirely excluded. Among the intermediates with complete degraded side chain, testosterone (**XI**), 1-dehydro-testosterone (**XII**), and compound **XIII** preliminarily identified as 9α-hydroxy-testosteronewere revealed.

It should be noted that testosterone, AD, and ADD have been specified as central intermediates in the steroid degradation pathway for many microorganisms and the steroid core destruction may proceed through 9α-hydroxy-ADD [1,21]. On the other hand, the formation of 9α-hydroxy-1-dehydrotestosterone (**XV**) as the intermediate of the 9(10)-seco pathway cannot be excluded even though its formation had not been reported yet. This assumption is in accordance with higher activity of KshA homologs toward testosterone as compared to AD, thus, confirming that the presence of 17-hydroxyl group does not prevent 9α-hydroxylation [54]. On the other hand, 1(2)-dehydrosteroids are generally more preferable substrates for KshA as compared with their 1(2)-saturated analogs [54], thus, allowing to propose 9α-hydroxy-1(2)-dehydro-testosterone as the intermediate for the 9(10)-seco pathway.

Destruction of steroid core at the early steps of cholesterol pathway, i.e., before the complete removal of the side chain at C-17, cannot be fully excluded either. The route could include B-ring rupture of cholesta-1,4-dien-3-one (**VI**) or 3-oxo-cholesta-1,4-dien-26-oic acid (**IX**) at the C9(10)-position due to the activity of 9α-hydroxylase (KshAB). The ortholog of *kshA* encoded by the gene *KR76_14170* (Table 3) was induced by both cholesterol and LCA. This assumption is in correspondence with the independency of the side chain degradation and steroid core oxidation, and generally correlates with the fast rate of cholesterol consumption and small amounts of the intermediates observed.

### 4.3. Main Steps of LCA Oxidative Destruction and Cholic Acid Interconversion Genes

**Figure 7:**
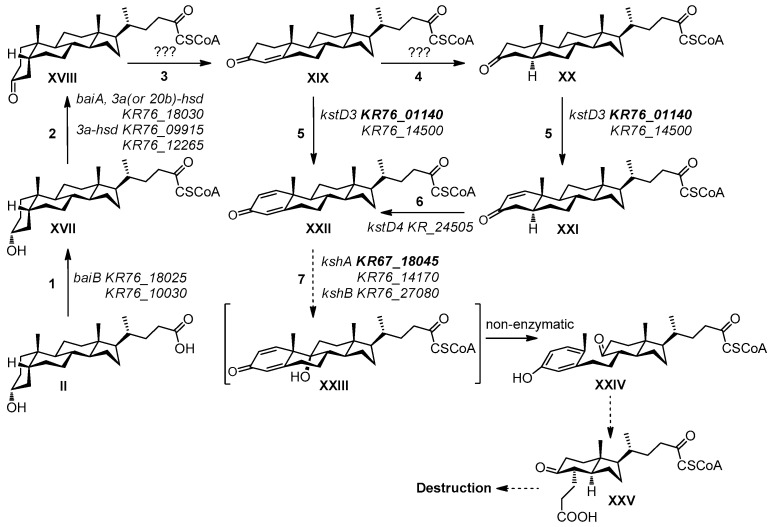
Scheme of the lithocholic acid bioconversion by *N. simplex* VKM Ac-2033D. Substances: **II**-Lithocholic acid, **XVII**-Lithocholanoyl-CoA, **XVIII**-3-Oxo-5β-cholan-24-oyl-CoA, **XIX**-3-Oxo-chol-4-en-24-oyl-CoA, **X** -3-Oxo-5α-cholan-24-oyl-CoA, **XXI**-3-Oxo-5α-chol-1-en-24-oyl-CoA, **XXII**-3-Oxo-chola-1,4-dien-24-oyl-CoA, **XXIII**-9α-Hydroxy-3-oxo-chola-1,4-dien-24-oyl-CoA, **XXIV**-3-Hydroxy-9-oxo-9,10-seco-chola-1,3,5(10)-trien-24-oyl-CoA, **XXV**-4*α*-(2-Carboxyethyl)-5-oxo-7a*β*,*γ*-dimethyl-3a*α*-hexahydroindane-1*β*-butyroyl-CoA. Biochemical reactions: 1—Ligation with CoA-SH,2—3α-Hydroxyl group dehydrogenation,3—3-Oxo-5β-cholan-24-oic acid 4(5)-dehydrogenation,4—3-Oxo-4-ene-steroids 4(5α)-reduction,5—3-Oxo-5α- or 3-oxo-4(5)-ene-cholan-24-oic acids 1(2)-dehydrogenation,6—3-oxo-1(2)-ene-cholan-24-oic acid 4(5)-dehydrogenation,7—9α-Hydroxylation. Locus tags in *N. simplex* are predicted, bold locus tags—genes that induced only by LCA.

Major reactions of LCA degradation by *N. simplex* were proposed based on cholic acid bioconversion by other actinobacteria such as *Corynebacterium* (*Arthrobacter*) *simplex* I.F.O. 3550 [55], and *R. jostii* RHA1 [14] (Figure 7). 

In order to predict the capability of the strain to transform or degrade cholic acids, the genes orthologous to those related to the bile acid metabolism in different bacteria (both soil-dwelling aerobic and human intestinal anaerobic bacteria) were considered (Table 4). The major bile salt modifications by intestinal bacteria include deconjugation, oxidation of hydroxy groups at C-3, C-7, and C-12, and C-7(α/β)-dehydroxylation. Soil actinobacteria such as *Rhodococcus* and *Nocardioides* species degrading cholic acids via the 9(10)-*seco* pathway might also contain orthologs of individual genes of bile acid interconversion.

Certain bile salt transporters were described in bacteria [59,61,62,63,64,65]. However, no orthologs of them were activated in *N. simplex* VKM Ac-2033D genome under the conditions used. The Mce4 system is known to provide steroid transport into actinobacteria cells [56]. In *N. simplex,* the *Mce*-genes *KR76_12195 - KR76_12230* could be involved in steroid transport. Enhanced expression of this operon was observed in response to both LCA and cholesterol (Table 4). This finding differs from that reported for *R. jostii* RHA1 where the Mce4 proteins were found only in the cholesterol grown cells [64] while cholate uptake is attributed to the channel-forming porins such as RjpA [65] and systems CamM and CamABCD [64]. Although the specific induction of *KR76_18015* (a permease) by LCA could also support the existence of a specific transport system for bile acids in *N. simplex*.

The enzymes that accounted for the alteration of the A/B ring juncture to form 3-keto-4-ene structure could be KstD4 coded by *KR76_24505* as well as 3α-HsDand other dehydrogenases revealed in the *N. simplex* genome that was suggested, but the clear insight requires a special deep investigation. 

It is well established that the initial step in the bacterial degradation of bile acids is the oxidation of the 3α-hydroxyl group catalyzed by 3α-hydroxysteroid dehydrogenases (3α-Hsd) (see Reference [2] for review). The enzymes such as HsdA from proteobacterium *Comamonas testosteroni* TA441 [51] or dual-function 3α,20β-hydroxysteroid dehydrogenase (3α,20β-Hsd) from the aerobic actinobacteria *Streptomyces hydrogenans* [66] have been shown to relate to the short chain dehydrogenase/reductase superfamily. The 3α-Hsd BaiA from anaerobic bacterium *Clostridium scindens* encoded by *HDCHBGLK_01433* was shown to act both on free bile salts and the corresponding CoA-esters [59]. Among the LCA-inducible genes in *N. simplex*, the genes *KR76_18030* and *KR76_18055* located in cluster E are the orthologs of the *R. jostii* RHA1 genes related to the bile acid catabolism with the only one annotated as *3α-hsd*. The gene *KR76_18030* looks as the most likely candidate that encodes 3α-Hsd in *N. simplex*. 

The orthogroup to which the *C. scindensHDCHBGLK_01433* belongs is the largest one that contains several dozen dehydrogenases. In *N. simplex*, two genes from this orthogroup were induced by LCA, namely, *KR76_12265* (3α-(or 20β)-hydroxysteroid dehydrogenase) and *KR76_18030* (3α-hydroxysteroid dehydrogenase). The latter gene increased its expression only in response to LCA, but not to cholesterol. Product of *KR76_12265* (cluster B) presumably might be active toward a wide range of steroid substrates while *KR76_18030* can play a role in cholate metabolism as 3α-hydroxysteroid dehydrogenase. Two other 3α-(or 20β)-hydroxysteroid dehydrogenases from this orthogroup, namely, *KR76_13560* and *KR76_25085,* did not enhance their expression under the conditions used. In addition, the genes annotated as 3α-hydroxysteroid dehydrogenases were also found in another orthogroup: *KR76_18035* (did not change its expression) and *KR76_09915* (induced by both LCA and cholesterol).

Ligation to CoA-esters was shown to be the first step in activating cholic and chenodeoxycholic acids [59]. BaiB (*HDCHBGLK_01430*) has been identified as a bile acid CoA ligase in *C. scindens* strain VPI 12,708 [2,57,59]. CasG is a CoA-ligase ortholog from cholate cluster of *R. jostii* RHA1 [14]. Of 28 orthologs of *baiB* and *casG* in *N. simplex* genome, only *KR76_10030* was induced with both LCA and cholesterol while the expression of *KR76_18025* mainly enhanced in the presence of LCA. Putatively, one or both these genes can be involved in cholate side chain activation. 

The *baiCD* gene from *C. scindens* (*HDCHBGLK_01431*) encodes a steroid oxidoreductase specific for the CoA conjugates of 3-oxo-chol-4-en-oic and 3-oxo-chenodeoxychol-4-en-oic acids [59]. The BaiCD polypeptide shows considerable amino acid sequence identity with the Old Yellow Enzyme family, a putative NADH oxidase, several 2,4-dienoyl CoA reductases, and BaiH from *C. scindens* [67]. In *N. simplex*, *KR76_18020*, that is orthologous to the *casH* gene in *R. jostii*, satisfies this description [14]. It was induced by LCA, but not by cholesterol.

Bile acid 7α-dehydratase is encoded by the *baiE* gene (*HDCHBGLK_01431* in *C. scindens*). 7α-Dehydration of 3-oxo-chol-4-en-oic and 3-oxo-chenodeoxychol-4-en-oic acids results in the generation of the conjugated double bonds in the rings A and B to form stable 3-oxo-deoxychola-4,6-dien-oic and 3-oxo-lithochola-4,6-dien-oic acids, respectively. Though LCA does not contain a 7-hydroxyl group, the ortholog of *baiE* in *N. simplex* (*KR76_16145*) was induced by both cholesterol and LCA, while *KR76_18075* increased its expression only with LCA. The enzyme showed no activity with the 3-oxo-ursodeoxychol-4-en-oic acid bearing 7β-hydroxyl group [68]. For the latter molecule, the *baiI* gene likely encodes a bile acid 7β-dehydratase, but orthologs of *baiI* are not found in *N. simplex*.

In *N. simplex*, the expression of the genes coding for the enzymes annotated as 3-ketosteroid dehydrogenase (KstD) and 3-ketosteroid 9α-hydroxylase (KshAB) was highly enhanced in LCA plus the condition as compared to cholesterol plus the condition. In addition to *KR76_14500*, another *kstD3* ortholog *KR76_01140*, located out of the clusters, was induced by LCA (Table 4). Bioinformatics calculations did not reveal any transcriptional factor site for the latter gene. In the presence of LCA, induction of two *kshA* orthologs was observed. Except *KR76_14170*, the genes *KR76_18045* and *KR76_27080*, a *kshB* ortholog, activated their transcription in LCA plus the condition. *KR76_18045* has a calculated Lcin1-site in the 550 bp-region. *KR76_27080* located in cluster D has no calculated site for transcriptional factor binding. It seems that LCA activates several means for the initial steps of the core degradation.

As shown for several actinobacteria such as *R. jostii* RHA1, *Corynebacerium simplex* I.F.O. 3530 and *Arthrobacter simplex* IICB 227, the presence of hydroxyl groups at C7 or C12 does not prevent the core degradation via the 9(10)-*seco* pathway [14,69,70,71,72].

According to the set of predicted orhtologs and DEGs, it can be assumed that the strain of *N. simplex* VKM Ac-2033D might be able to utilize cholic acid, chenodeoxycholic acid, LCA, and their oxidized variants, but not bile acids with 7β-hydroxyl in the core. The results generally correspond to the previously reported data on the consumption of cholic acid and oxidation of deoxycholic acid by *N. simplex* [22] as well as the strain growth on LCA.

### 4.4. Regulation and Candidate Motifs for LCA Catabolism Transcriptional Factors

Most of the genes that activated transcription in response to LCA belonged to the previously predicted KstR and KstR2 regulons. However, the motifs for steroid catabolism transcriptional regulator were not known for certain DEGs including steroid catabolism orthologs. In this study, we predict a new candidate motif for LCA catabolism relying on the transcriptome studies.

Among KstR3 [14] orthologs in *N. simplex* VKM Ac-2033D genome, only the gene *KR76_18060* enhanced expression in response to LCA more than to cholesterol (*KR76_18040*, that is a possible ortholog of regulator *casA* from *R. jostii* RHA1, was not induced).

Calculations using DEG data demonstrated the presence of at least two motifs and two candidate transcriptional factors that could regulate cholate catabolism in this organism. Lcin1 was revealed in cluster E for the orthologs of cholate catabolism genes induced by LCA. Sites of Lcin1 were also revealed before operon *KR76_24170*-*KR76_24180* that were orthologs of HsaEGF [73] or TesEGF [74] involved in degradation of the A-ring after its cleavage in C6-moiety. Lcin2 was found for different genes, but some of these genes also belonged to other predicted regulons (KstR and KstR2). Thus, this motif seems to not be important for the studied processes.

Presumably, the transcriptional factors that are associated with the revealed motifs may be the *KR76_18060* (from cluster E) and *KR76_18605* (out of clusters) since only the genes annotated as transcriptional factors are induced in LCA plus conditions. However, this statement requires further research.

## 5. Conclusions

The specific features of steroid degradation by an industrially relevant strain of *Nocardioides simplex* VKM Ac-2033D using cholesterol and lithocholic acid as model substrates have been studied. A number of intermediates of sterol sidechain oxidation have been revealed, such as 3β-hydroxy-cholest-5-en-26-oic acid and 3-oxo-cholesta-1,4-dien-26-oic, that have not been reported earlier for *N. simplex* and related species.

Though the major degradation pathways of cholesterol and LCA are generally similar and include the 9(10)-*seco* pathway as a basic steroid core degradation route, the genetic control of the initial steps of steroid core and the side chain oxidation seems to be distinct for the different classes of steroids. As compared to cholesterol, LCA can induce an additional set of the genes, including those coding for Δ^1^-3-ketosteroid dehydrogenases and 3-ketosteroid 9α-hydroxylase. One can assume a parallel or an additional pathway of steroid core utilization.

The gene cluster mainly related to the catabolism of bile acids consisted of the genes that did not possess binding sites to the known steroid degradation regulators KstR and KstR2. Candidate motifs of transcription factors were calculated for this cluster. Based on the bioinformatic analysis, the activities toward different bile acids have been predicted that generally match the known experimental data. Functions of many orthologs of steroid catabolism genes have been predicted.

The findings of the study contribute to understanding bacterial metabolism of different steroids, diversity of the related gene networks involved, and their transcription regulation.

## Figures and Tables

**Table 1:** Steroid intermediates detected during cholesterol bioconversion by *N. simplex* VKM Ac-2033D.

Number	Name	Molecular Weight	Chemical Structure	Number	Name	Molecular Weight	Chemical Structure
**V**	Cholest-4-en-3-one	384	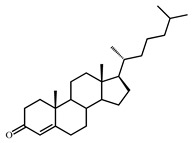	**VI**	Cholesta-1,4-dien-3-one	382	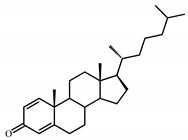
**VII**	26-Hydroxy-cholest-4-en-3-one	400	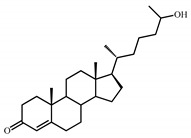	**VIII**	3-Oxo-cholest-4-en-26-oic acid	414	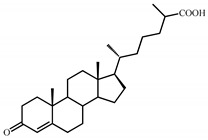
**IX**	3-Oxo-cholesta-1,4-dien-26-oic acid	412	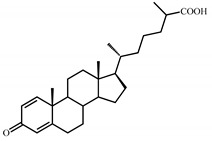	**X**	3β-Hydroxy-cholest-5-en-26-oic acid	416	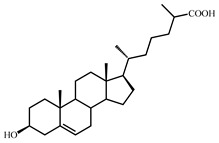
**XI**	Androst-4-en-17β-ol-3-one, Testosterone	288	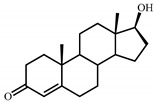	**XII**	Androsta-1,4-dien-17β-ol-3-one, 1-Dehydrotestosterone	286	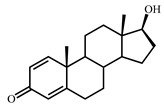
**XIII**	Androst-4-ene-9α,17β-diol-3-one, 9α-Hydroxy-testosterone(preliminarily determined)	304	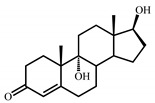				

**Table 2:** Differentially expressed genes in gene clusters.

Cluster	Locus Tags	Cholesterol				LCA				LCA/Cholesterol			
		I	Ave	Max	D	I	Ave	Max	D	I	Ave	Max	D
A	*KR76_14160*-*KR76_14505*	23	50	226	0	28	40	108	0	0			0
B	*KR76_12190*-*KR76_12345*	16	14	38	0	19	17	34	0	0			0
C	*KR76_25015*- *KR76_25200*	2	9	13	0	9	13	65	0	2	5	5	0
D	*KR76_27035*-*KR76_27130*	0			0	1	8	8	1	1	8	8	0
E	*KR76_17985*-*KR76_18075*	0			1	15	25	44	0	17	31	58	0
Out of clusters		86			19	84			20	24			19
Total		127			20	156			21	44			19

Cluster: cluster of genes to which the gene is assigned. Locus_tags: range of genes locus tags that are included in the cluster. Cholesterol: number of genes changing expression in response to cholesterol (in comparison with the control) with more than threefold changes and *q*-value less than 0.05. LCA: number of genes changing expression in response to LCA (in comparison with the control) with more than threefold changes and *q*-value less than 0.05. LCA/Cholesterol: number of genes changing expression in response to LCA in comparison with cholesterol with more than threefold changes and *q*-value less than 0.05. I: the number of genes that significantly (more than 3 times with *q*-value < 0.01) increased their expression. Ave: the average level of induction among the genes that significantly increased their expression in the cluster. Max: the maximum level of induction. D: the number of genes that significantly (more than 3 times with *q*-value < 0.01) reduced their expression.

**Table 3:** Genes involved in the initial steps of steroid oxidation in *N. simplex.*

Step	Gene Name	Annotation	Locus Tag	Cluster	Regulon	LCA	Chol	References
1	*cho*	cholesterol oxidase	*KR76_09550*		KstR	+	+	[39]
	*ksdI*, *ksi*	∆^5^-3-ketosteroid isomerase	*KR76_23530*		KstR		+	[40]
			*KR76_24125*		KstR	+	+	
2	*kstD3, tesH*	∆^1^-3-ketosteroid dehydrogenase with preference for the 1,2-saturated steroid substrates	*KR76_14500*	A	KstR	*+*	*+*	[41,42,43]
3, 4	*cyp125*	cytochrome P450 125	*KR76_14335*	A	KstR	+	+	[44]
6	*hsd4A*	17β-hydroxysteroid dehydrogenase or β-hydroxyacyl-CoA dehydrogenase	*KR76_12260*	B	KstR	*+*		[45]
7	*kshA*	3-ketosteroid 9α-hydroxylase, oxygenase subunit	*KR76_14170*	A	KstR	*+*	*+*	[46]
	*kshB*	3-ketosteroid 9α-hydroxylase, reductase subunit	*KR76_14160*	A	KstR			[46]

Step: number of oxidation step from Figure 6. Gene name: name of gene homolog in *Mycobacterium tuberculosis* H37Rv or in *Rhodococcus jostii* RHA1. Annotation: function of enzyme. Locus_tag: locus_tag of ortholog in *N. simplex* VKM Ac-2033D genome that is a candidate gene for this function. Cluster: cluster of genes to which the gene is assigned (see Figure 3). Regulon: repressor whose binding site is predicted in the promoter of the gene operon. LCA: gene is induced by LCA (+). Chol: gene is induced by cholesterol (+). References: reference source where information about the gene function was taken.

**Table 4:** Candidate genes for initial steps of cholate degradation and cholic acids interconversion.

Step	Enzyme Annotation	Gene Name	*C. scindens*	*R. jostii*	*N. simplex*	Cluster	Regulon	LCA	Chol	Reference
	transporter	*mce*			*KR76_12195- KR76_12230*	B	KstR	+	+	[56]
1	bile acid CoA ligase	*baiB*	*HDCHBGLK_01430*	*casG*	*KR76_10030*		KstR	+	+	[57]
					*KR76_18025*	E		+		
2	3α-hydroxysteroid dehydrogenase	*baiA*	*HDCHBGLK_01433*		*KR76_18030*	E		+		[58]
	3α-(or 20β)-hydroxysteroid dehydrogenase				*KR76_12265*	B	KstR	+	+	
	3α-hydroxysteroid dehydrogenase				*KR76_09915*	out	KstR	+	+	
	short-chain alcohol dehydrogenase				*KR76_18055*	E		+		[59]
4	Δ^4^-(5α)-3-ketosteroid dehydrogenase	*kstD4, tesI*			*KR76_24505*	out	KstR	*+*	*+*	[47,60]
5	Δ^1^-3-ketosteroid dehydrogenases	*kstD3, tesH*			*KR76_01140*			*+*		[41,43]
					*KR76_14500*	A	KstR	*+*	*+*	
6	3-ketosteroid 9α-hydroxylase, oxygenase subunit	*kshA*			*KR76_14170*	A	KstR	*+*	*+*	[46]
					*KR76_18045*	E		*+*		
	3-ketosteroid 9α-hydroxylase, reductase subunit	*kshB*			*KR76_14160*	A	KstR			
					*KR76_27080*	D		*+*		
	oxidoreductase	*baiCD*		*casH*	*KR76_18020*			+		[59]
	bile acid 7α-dehydratase	*baiE*	*HDCHBGLK_01432*		*KR76_16145*		KstR	+	+	[59]
					*KR76_18075*	E		+		
	flavin-dependent oxidoreductase			*ro05794*	*KR76_18065*	E			+	[14]

Step: number of catabolism step from Figure 7. Enzyme annotation: function of enzyme. Gene name: name of gene homolog. *C. scindens*: locus tag of homolog in *Clostridium scindens*. *R. jostii*: name or locus tag of homolog in *R. jostii* RHA1. *N. simplex*: locus tag of ortholog in *N. simplex* VKM Ac-2033D genome that is candidate gene for this function. Cluster: cluster of genes to which the gene is assigned (see Figure 3). Regulon: repressor whose binding site is predicted in the promoter of the gene operon.LCA: gene is induced by LCA (+). Chol: gene is induced by cholesterol (+). References: reference source where information about the gene function was taken.

## Supplementary Materials

The following are available online at www.mdpi.com/xxx/s1/. Figure S1: Change in content of the intermediates during cholesterol biotransformation by *N. simplex* VKM Ac-2033D. (**a**) 3-Oxo-cholest-4-en-26-oic acid and 3-oxo-cholesta-1,4-dien-26-oic acid (TLC, visualization under UV, 254 nm). The reference compound is cholest-4-en-3-one. (**b**) 3β-Hydroxy-cholest-5-en-26-oic acid visualization after phosphomolybdic acid (TLC, visualization with phosphomolybdic acid staining). The reference compound is cholesterol. **C**. LCA decrease during its bioconversion by *N. simplex* (visualization with MnCl_2_ staining). The reference compound is 3-oxo-deoxycholic acid. Figure S2: LOGO of transcriptional regulator motifs. (**a**) Motif for Lcin1. (**b**) Motif for Lcin2. Table S1: Real-time PCR. Table S2: List of orthogroups. Table S3: Steroid intermediates and full characteristics. Table S4: Read counts. Table S5: DEGs, operons, and predicted regulons.
