# Supplementary material for: Genome-Wide Transcriptome Profiling Provides Insight on Cholesterol and Lithocholate Degradation Mechanisms in Nocardioides simplex VKM Ac-2033D
Source: Genes (Basel). 2020 Oct 20;11(10):1229. doi: 10.3390/genes11101229 (PMC7593942; doi:10.3390/genes11101229)
Supplement: Supplementary file 1 [file genes-11-01229-s001.zip › Supplementary_table_S1._Real-time_qPCR_v.4.docx]

**Supplementary table S1 Real-time qPCR**

Primers for real-time Q-PCR

| Gene | F | R | Tm | Length |
| --- | --- | --- | --- | --- |
| *IF 1 KR76_21135* | ATGTGAGCCAGGACCTTGTG | ATGGCGAAGAAAGAAGGCGT | 60 | 110 |
| *gyrA KR76_00045* | ACTGAAACCCAGAGCAACCT | TGGCGTAGTCGATGTAGGAG | 60 | 100 |
| *petA KR76_10970* | GCTCATAGTAGTCACGCTCCC | ACTCGAAGATCTGCACCCAC | 60 | 246 |
| *mceB KR76_12210* | CAAGGACTGGATGACCGACC | TCCTCCCGGTTCTCCTTCTT | 60 | 183 |
| *ltp3 KR76_14285* | TGATGATGCCCGAGCTCTAC | ACGGTCAGCACCTTCTTGTG | 60 | 151 |
| *ltp4 KR76_14280* | AGAAGGACATCGGCTTCTGG | ACCTCACCGGTCTGGATCTT | 60 | 178 |

Expression changes (2^-ΔΔCt^) for target genes

| Gene | Cholesterol induction | LCA induction |
| --- | --- | --- |
| *ltp3 KR76_14285* | 32 | 22 |
| *ltp4 KR76_14280* | 14 | 7 |
| *mceB KR76_12210* | 6 | 4 |
