# Supplementary figures and images for: Genome-Wide Transcriptome Profiling Provides Insight on Cholesterol and Lithocholate Degradation Mechanisms in Nocardioides simplex VKM Ac-2033D
Source: Genes (Basel). 2020 Oct 20;11(10):1229. doi: 10.3390/genes11101229 (PMC7593942; doi:10.3390/genes11101229)

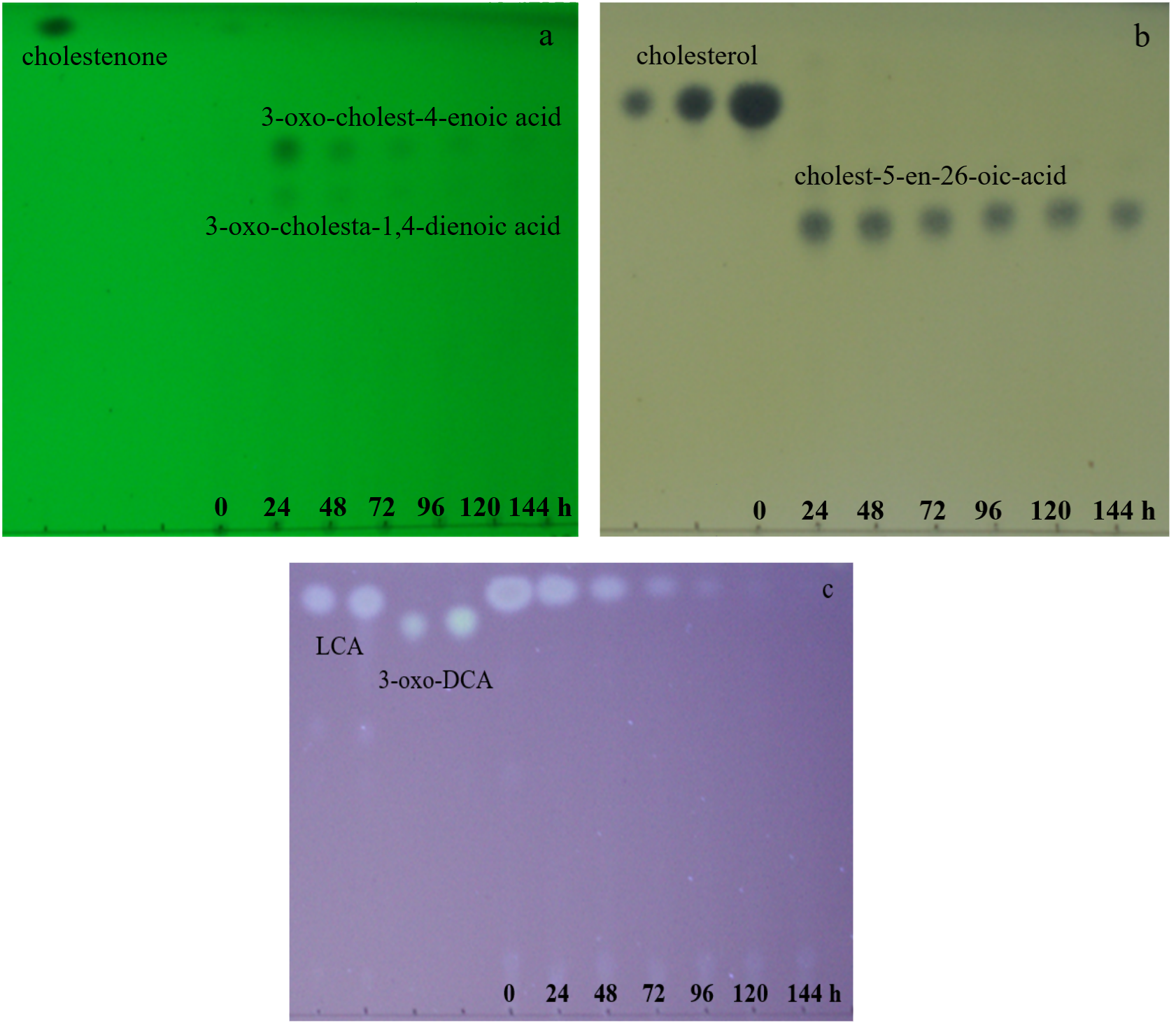

Supplement: Supplementary file 1 [file genes-11-01229-s001.zip › Supplementary_Figure_S1_v4_G.tiff]

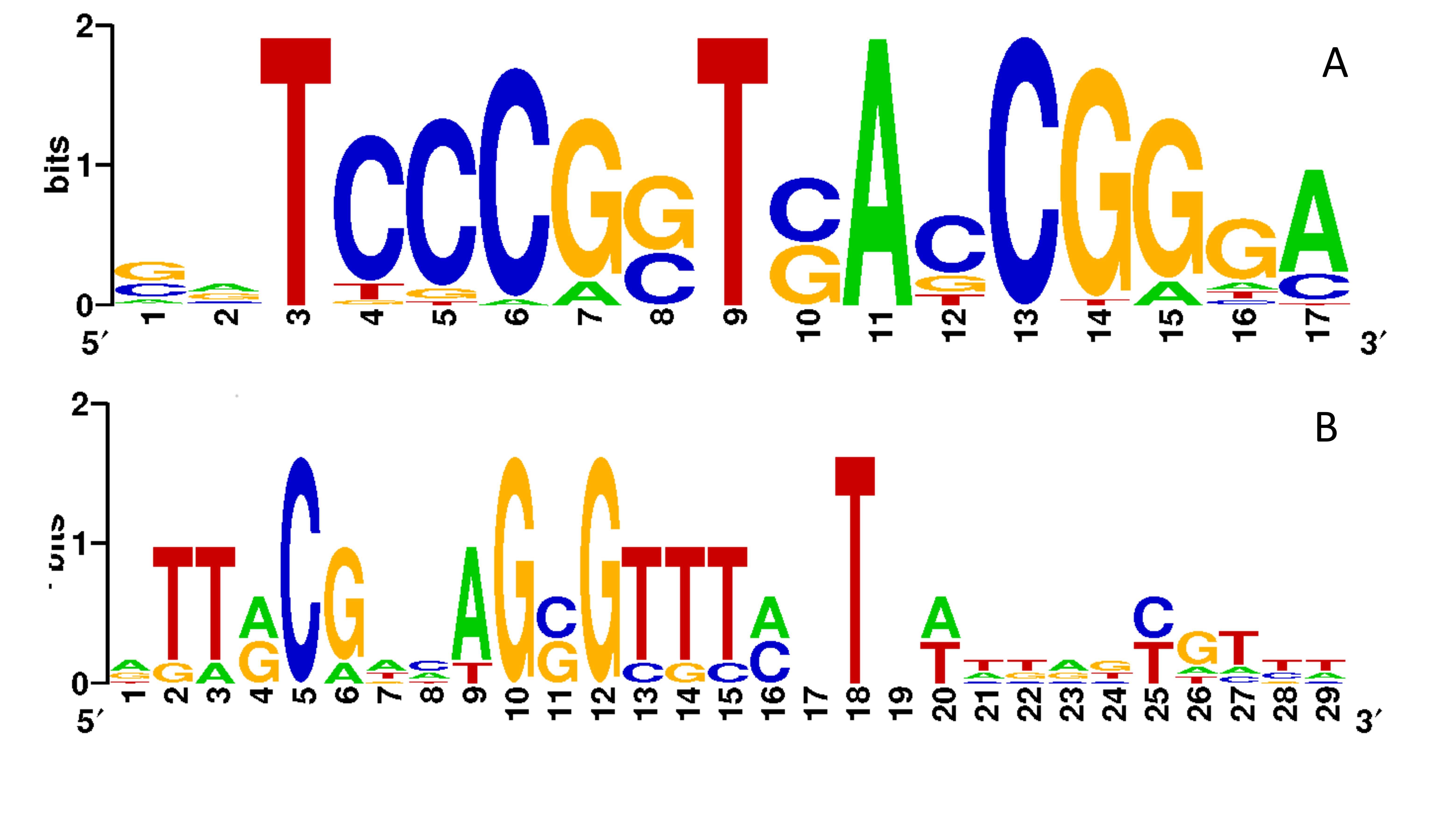

Supplement: Supplementary file 1 [file genes-11-01229-s001.zip › Supplementary_Figure_S2_Motifs_v4_G.tiff]
