# Supplementary material for: Genome-Wide Transcriptome Profiling Provides Insight on Cholesterol and Lithocholate Degradation Mechanisms in Nocardioides simplex VKM Ac-2033D
Source: Genes (Basel). 2020 Oct 20;11(10):1229. doi: 10.3390/genes11101229 (PMC7593942; doi:10.3390/genes11101229)
Supplement: Supplementary file 1 [file genes-11-01229-s001.zip › Supplementary_Table_S3._v4_G.doc]

**Supplementary Table S3 Sterol intermediates detected during cholesterol bioconversion by *N. simplex* VKM Ac-2033D**

| **Compound** | **Mw** | **Structure** | **Rt, System**  **acetonitrile:*iso*-propanol:water (50:45:5)^1,2^** | **Rt, System acetonitrile:water:acetic acid (60:40:0.01)^1,2^** | **Rf, System**  **benzene:acetone (4:1)** |
| --- | --- | --- | --- | --- | --- |
| Cholest-4-en-3-one | 384 |  | 9.2 | n.d. | 0.89 |
| Cholesta-1,4-dien-3-one,  1-Dehydro-cholestenone | 382 |  | 7.3 | n.d. | 0.87 |
| 26-Hydroxy-cholest-4-en-3-one | 400 |  | 3.88 | 80.2 | 0.64 |
| 3-Oxo-cholest-4-en-26-oic acid | 414 |  | 3.69 | 51.7 | 0.65 |
| 3-Oxo-cholesta-1,4-dien-26-oic acid | 412 |  | 3.4 | 32.6 | 0.56 |
| 3β-Hydroxy-cholest-5-en-26-oic acid | 416 |  | 3.65 | 45.9 | 0.48 |
| Androst-4-en-17β-ol-3-one,  Testosterone | 288 |  | n.d. | 5.25 | 0.48 |
| Androsta-1,4-dien-17β-ol-3-one,  1-Dehydrotestosterone | 286 |  | n.d. | 4.37 | 0.38 |
| Androst-4-en-9α,17β-diol-3-one,  9α-Hydroxy-testosterone  (preliminary determined) | 304 |  | n.d. | n.d. | 0.38 |

^1^ 3β-hydroxy-5-en steroids under UV-detection (200 nm);

^2^ 3-keto-4-en steroids under UV-detection (240 nm);

n.d. – not determined in this system

Cholest-4-en-3-one (V), cholesta-1,4-dien-3-one (VI), 26-hydroxy-cholest-4-en-3-one (VII) have been detected in the samples taken after 6 h bioconversion,

3-Oxo-cholest-4-en-26-oic acid (VIII), 3-oxo-cholesta-1,4-dien-26-oic acid (IX), 3β-hydroxy-cholest-5-en-26-oic acid (X), androst-4-en-17β-ol-3-one (XI), androsta-1,4-dien-17β-ol-3-one (XII) were detected in the samples taken after 24 h bioconversion.

## **HPLC-profiles for mobile phase acetonitrile:2-propanol:water (50:45:5 v/v/v)**

Cholesterol (standard)

Cholest-4-en-3-one (cholestenone) (standard)

Cholest-4-en-3-one (**V**)

Cholesta-1,4-dien-3-one (**VI**)

26-Hydroxy-cholest-4-en-3-one (**VII**)

3-Oxo-cholest-4-en-26-oic acid (**VIII**)

3-Oxo-cholesta-1,4-dien-26-oic acid (**IX**)

3β-Hydroxy-cholest-5-en-26-oic acid (**X**)

**HPLC-profiles for mobile phase acetonitrile:water:acetic acid (60:40:0.01 v/v/v)**

26-Hydroxycholest-4-en-3-one (**VII**)

3-Oxo-cholest-4-en-26-oic acid (**VIII**)

3-Oxo-cholesta-1,4-dien-26-oic acid (**IX**)

3β-Hydroxy-cholest-5-en-26-oic acid (**X**)

Androst-4-en-17β-ol-3-one (testosterone) (standard)

Androst-4-en-17β-ol-3-one (testosterone) (**XI)**

Androsta-1,4-dien-17β-ol-3-one (1-dehydrotestosterone) (standard)

Androsta-1,4-dien-17β-ol-3-one (1-dehydrotestosterone) (**XII**)

**Mass spectra [M+H]**

Cholest-4-en-3-one (**V**)

Cholest-4-en-3-one (**V**) (collision energy 25 eV)

Cholesta-1,4-dien-3-one (**VI**)

Cholesta-1,4-dien-3-one (**VI**) (collision energy 26 eV)

3-Oxo-cholest-4-en-26-oic acid (**VIII**)

3-Oxo-cholest-4-en-26-oic acid (**VIII**) (collision energy 23 eV)

3-Oxo-cholesta-1,4-en-26-oic acid (**IX)**

3-Oxo-cholesta-1,4-en-26-oic acid (**IX)** (collision energy 21 eV.)

26-Hydroxycholest-4-en-3-one (**VII**)

26-Hydroxycholest-4-en-3-one (**VII**) (collision energy 24 eV)

Androst-4-en-17β-ol-3-one (testosterone) (**XI)**

Androst-4-en-17β-ol-3-one (testosterone) (**XI) (**collision energy 23 eV)

1(2)-Dehydro-testosterone (**XII**)

1(2)-Dehydro-testosterone (**XII**) **(**collision energy 26 eV)

9α-Hydroxy-testosterone **(XIII**) **(**collision energy 22 eV)

**Mass-spectra HRMS-ESI (*m/z*): [M-H]^+^**

HRMS-ESI (*m/z*): [M-H]^+^ calcd for C_27_H_41_O_3_ 413,3056; found 413,3059.

3-Oxocholest-4-en-26-oic acid (**VIII**).

HRMS-ESI (*m/z*): [M-H]^+^ calcd for C_27_H_39_O_3_ 411,2899; found 411, 2903.

3-Oxocholesta-1,4-dien-26-oic acid (**IX**).

HRMS-ESI (*m/z*): [M-H]^+^ calcd for C_27_H_43_O_3_ 415,3212; found 415,3217.

3β-Hydroxy-cholest-5-en-26-oic acid (**X**)

**^1^H- and ^13^C-NMR spectra**

3-Oxo-cholest-4-en-26-oic acid (**VIII**), ^1^H-NMR

^1^H-NMR (CDCl_3_) δ: 5.73 (br. s., 1H, 4-H), 1.18 (s, 3H, 19-CH_3_), 1.17 (d, *J* = 7.0 Hz, 3H, 27-CH_3_), 0.91 (d, *J* = 6.5 Hz, 3H, 21-CH_3_), 0.70 (s, 3H, 18-CH_3_).

3-Oxo-cholest-4-en-26-oic acid (**VIII**), ^13^C-NMR

^13^C-NMR (CDCl_3_) δ: 199.9 (C-3), 182.3 (C-26), 171.9 (C-5), 123.7 (C-4), 56.0, 55.8, 53.8, 42.4, 39.6, 39.2, 38.6, 35.63, 35.56, 33.92, 33.86, 32.9, 32.0, 28.1, 24.1, 23.6, 21.0, 18.5, 17.3, 16.7, 11.9.

3-Oxo-cholesta-1,4-dien-26-oic acid (**IX**), ^1^H-NMR

^1^H-NMR (CDCl_3_) δ: 7.06 (d, *J* = 10.1 Hz, 1H, 1-H), 6.24 (dd, *J* = 1.9, 10.1 Hz, 1H, 2-H), 6.08 (br. s., 1H. 4-H), 1.23 (s, 3H, 19-CH_3_), 1,17 (d, *J* = 7.0 Hz, 3H, 27-CH_3_), 0.91 (d, *J* = 6.5 Hz, 3H, 21-CH_3_), 0.73 (s, 3H, 18-CH_3_).

3-Oxo-cholesta-1,4-dien-26-oic acid (**IX**), ^13^C-NMR

^13^C-NMR (CDCl_3_) δ: 186.6 (C-3), 182.3 (C-26), 169.8 (C-5), 156.3 (C-1), 127.4 (C-2), 123.7 (C-4), 56.0, 55.4, 52.3, 43.7, 42.6, 39.4, 39.2, 35.6, 35.5, 35.4, 33.9, 33.7, 32.9, 28.1, 24.4, 23.6, 22.8, 18.6, 18.5, 16.7, 12.0.

3β-Hydroxy-cholest-5-en-26-oic acid (**X**), ^1^H-NMR

^1^H-NMR (CD_3_OD) δ: 5.33 (d, *J* = 5.2 Hz, 1H, 6-H), 3.39 (m, 1H, 3α-H), 1.12 (d, *J* = 7.0 Hz, 3H, 27-CH_3_), 1.01 (s, 3H, 19-CH_3_), 0.93 (d, *J* = 6.5 Hz, 3H, 21-CH_3_), 0.71 (s, 3H, 18-CH_3_).

3β-Hydroxy-cholest-5-en-26-oic acid (**X**), ^13^C-NMR

^13^C-NMR (CD_3_OD) δ: 180.8 (C-26), 142.2 (C-5), 122.5 (C-6), 72.4 (C-3), 58.2, 57.5, 51.7, 43.5, 43.0, 41.2, 40.7, 38.6, 37.7, 37.0, 35.4, 33.3, 33.0, 32.3, 29.3, 25.3, 24.8, 22.2, 19.9, 19.2, 17.6, 12.4.
