# Supplementary material for: Genome-Wide Transcriptome Profiling Provides Insight on Cholesterol and Lithocholate Degradation Mechanisms in Nocardioides simplex VKM Ac-2033D
Source: Genes (Basel). 2020 Oct 20;11(10):1229. doi: 10.3390/genes11101229 (PMC7593942; doi:10.3390/genes11101229)
Supplement: Supplementary file 1 [file genes-11-01229-s001.zip › Supplementary_Table_S4._Sequencing_data_v4_G.doc]

Supplementary Table S4 Read counts

| **Sample** | **Number of reads mapping to CDS** |
| --- | --- |
| Control_MCD_repl1 | 409 054 |
| Control_MCD_repl2 | 1 060 989 |
| Cholesterol_MCD_repl1 | 2 851 365 |
| Cholesterol_MCD_repl2 | 6 141 824 |
| Lithocholate_MCD_repl1 | 1 492 098 |
| Lithocholate_MCD_repl2 | 5 594 141 |

Reads in SRA: https://www.ncbi.nlm.nih.gov/bioproject/PRJNA350502
